# Quantification of Poly(vinyl chloride) Microplastics *via* Pressurized Liquid Extraction and Combustion Ion Chromatography

**DOI:** 10.1021/acs.est.2c06555

**Published:** 2023-03-14

**Authors:** Jan Kamp, Georg Dierkes, Peter Nikolaus Schweyen, Arne Wick, Thomas A. Ternes

**Affiliations:** Federal Institute of Hydrology, Am Mainzer Tor 1, 56068 Koblenz, Germany

**Keywords:** plastics, environmental pollution, solvent extraction, sediments, suspended matter, chlorine-containing polymers

## Abstract

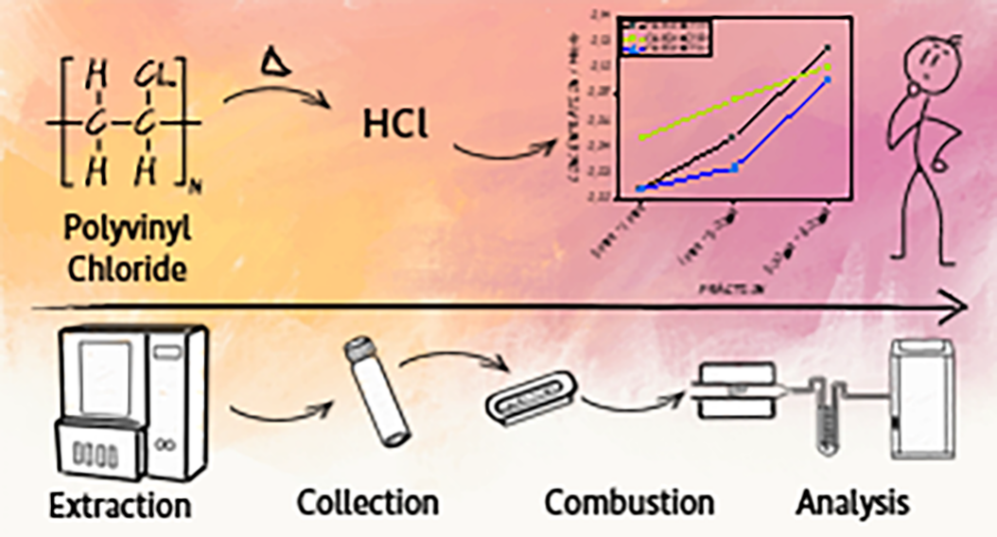

A reliable analytical method has been developed to quantify poly(vinyl chloride) (PVC) in environmental samples. Quantification was conducted *via* combustion ion chromatography (C-IC). Hydrogen chloride (HCl) was quantitatively released from PVC during thermal decomposition and trapped in an absorption solution. Selectivity of the marker HCl in complex environmental samples was ensured using cleanup *via* pressurized liquid extraction (PLE) with methanol at 100 °C (discarded) and tetrahydrofuran at 185 °C (collected). Using this method, recoveries of 85.5 ± 11.5% and a limit of quantification down to 8.3 μg/g were achieved. A variety of hard and soft PVC products could be successfully analyzed *via* C-IC with recoveries exceeding >95%. Furthermore, no measurable overdetermination was found for various organic and inorganic matrix ingredients, such as sodium chloride, sucralose, hydroxychloroquine, diclofenac, chloramphenicol, triclosan, or polychlorinated biphenyls. In addition, sediments and suspended particular matter showed PVC concentrations ranging up to 16.0 and 220 μg/g, respectively. However, the gap between determined polymer mass and particle masses could be significant since soft PVC products contain plasticizers up to 50 wt %. Hence, the results of the described method represent a sum of all chlorine-containing polymers, which are extractable under the chosen conditions.

## Abstract 2

The presented method offers an opportunity for PVC quantification to counteract overdeterminations caused by matrix effects.

## 1 Introduction

Since the discovery of plastics up to the present day, the number and variety of these materials are steadily increasing due to their manifold applications. To date, plastics are used in almost all areas, such as packaging, consumer goods, construction materials, and many other fields.^1,2^ As a consequence, they are currently detected in all kinds of environmental matrices.^3,4^ In general, detected plastics are divided into two types: macroplastics and microplastics. Microplastics are defined as small plastic particles with a diameter of less than 5 mm, while macroplastics are particles of 5 mm diameter and above.^5^ Microplastic particles enter the environment either directly (primary microplastic) or are formed through the decomposition of macroplastics due to physical processes, weathering, or sunlight (secondary microplastics).^6−8^ Detailed quantitative studies are needed to identify sources, distribution routes, and the loads released. Poly(vinyl chloride) (PVC) is an important synthetic polymer accounting for *ca.* 9.6% of global plastic production.^2^ Since PVC is mainly used as a construction material for pipes, the polymer is particularly relevant for microplastic research in environmental analysis.^2^

In recent years, different optical analytical methods, such as microscopy, Raman spectroscopy, or Fourier transform infrared spectroscopy, have been used for the identification of various types of plastic particles.^9−12^ Also, analytical methods using mass spectrometry in environmental samples have been developed for different polymer types, such as polyethylene (PE), polypropylene (PP), polystyrene (PS), poly(vinyl chloride) (PVC), poly(ethylene terephthalate) (PET), polycarbonate (PC), or poly(methylmethacrylate) (PMMA) as well as their additives.^13−16^ Primarily, pyrolysis or thermogravimetric analysis (TGA) has been coupled with gas chromatograpy/mass spectrometry (GC-MS).^16−22^ However, these thermoanalytical methods are still under development and suffer from matrix interferences and false-positive results.^13,16,23−25^ For instance, natural compounds from coniferous trees could lead to PE overquantifications.^26^ Furthermore, in the case of PS, Fischer et al. showed possible overdeterminations by phenylalanine from proteins during pyrolysis-GC-MS.^27^ Regarding PVC, in the current literature, using pyrolysis or TGA, PVC, benzene, or naphthalene has often been used as a quantifier.^6,19,25,27^ However, both pyrolysis products are very unspecific as they can also be generated during the pyrolysis of other synthetic polymers, such as PET or organic substances.^25−27^ Therefore, severe matrix effects leading to an overdetermination of PVC cannot be excluded. Another challenge for the quantification of microplastics is the matrix effects of different matrix ingredients.^25,29^ Hence, there is still a lack of an alternative analytical method, enabling the specific and reliable quantification of PVC in environmental samples. A unique feature of PVC and related polymers, such as poly(vinylidene chloride) or chlorinated PE, compared to other polymers, is chlorine. During pyrolysis, chlorine is eliminated in the form of hydrogen chloride (HCl), making it a promising marker for quantification of chlorinated polymers mainly PVC. However, due to its high volatility, polarity, and acidity, HCl is hardly analyzable *via* GC-MS. An alternative is the trapping of HCl in an absorption solvent and an analysis *via* ion chromatography.^30−32^

The goal of our study was to develop a reliable and sensitive analytical method to quantify PVC in environmental samples down to the lower μg/g range *via* combustion ion chromatography (C-IC) with conductivity detection. The newly developed method was utilized for PVC quantification in suspended particulate matter (SPM) and sediments.

## 2 Methods

### 2.1 Extraction

For sample extraction, methanol from Merck (MeOH, liquid chromatography–mass spectrometry (LC-MS) grade, Darmstadt, Germany) and tetrahydrofuran from Sigma-Aldrich (THF, unstabilized, Schelldorf, Germany) were used. Extraction cells were filled up with calcined sea sand. A SpeedExtractor E-914 system (BÜCHI Labortechnik GmbH, Essen, Germany) was used for all extractions. Every sample was extracted with MeOH at 100 °C and 100 bar as cleanup (discarded), followed by the extraction with THF at 185 °C and 100 bar, according to Dierkes et al.^13^ The THF extract was collected in 60 mL vials each filled with 200 mg of silica gel 60 (70–270 mesh, Machery-Nagel, Düren, Germany) as the collecting medium. After extraction, THF was evaporated, and the remaining silica gel was then ground and homogenized by a mortar.

### 2.2 Calibration and Recoveries

For quantification and recovery experiments, additive-free PVC was purchased from PyroPowders.de (Erfurt, Germany) and dissolved in THF. The calibration series were set in a range from 0.005 to 5.0 mg/g. For this, PVC was dissolved in THF at room temperature and a serial dilution was prepared, which was then directly placed onto C-IC boats and fed into the combustion ion chromatography system (C-IC) for determination of the recovery. Another calibration was performed using the extraction method mentioned above. All samples were prepared in triplicate.

To determine extraction recoveries, samples (*n* = 8) spiked with a concentration of 2.5 mg/g PVC in calcined sea sand (2 h, 700 °C) were extracted and analyzed as described above. PVC quantification here was performed using calibration *via* direct PVC combustion excluding the extraction step. To evaluate matrix effects, multiple extractions were carried out using 1.0 g of sediment from the Rhine harbor of Ehrenbreitstein in Koblenz (EBS), unspiked (*n* = 8) and spiked with 1.0 mg/g PVC (*n* = 8). PVC quantification here was done using calibration including the extraction step.

### 2.3 Combustion Ion Chromatography

All C-IC measurements were carried out using an ASC-240S autosampler and an AQF-2100H combustion oven (Mitsubishi Chemical Analytech, Yamato-shi, Japan). For absorption, a GA-210 absorption unit (Mitsubishi Chemical Analytech) was used. Furthermore, an 881 Compact IC pro (Metrohm, Filderstadt, Germany) with a conductivity detector was used for the detection of chloride. A scheme of the whole setup is shown in the Supporting Information (Figure S1). Table 1 comprises the detailed experimental setup and parameters used for each measurement.

**Table 1:** Experimental Parameters and Instrumental Parts of the C-IC System

instrumental parameters	value
combustion	
instrument	ASC-240S (autosampler), AQF-2100H (combustion)
furnace temperature	900–1000 °C
combustion time	18 min
carrier gas	200 mL/min Ar (5.0, Linde GmbH, Pullach, Germany)
	400 mL/min O_2_ (5.0, Linde GmbH)
absorption	
instrument	GA-210
absorption solution	300 mg/L H_2_O_2_, 5 mg/L phosphate, MilliQ water
absorption solution volume	10 mL
ion chromatography	
instrument	881 compact IC pro
column	Metrosep A Supp 5–150/4.0
mobile phase	3.2 mmol/L Na_2_CO_3_; 1.0 mmol/L NaHCO_3_; isocratic
suppressor	250 mmol/L H_3_PO_4_
flow rate	0.7 mL/min
oven temperature	45 °C
injection volume	100.0 μL
detector	conductivity detector

For all measurements, 20 mg of each silica gel obtained from THF extracts was weighed onto ceramic boats and were combusted at about 1000 °C for 18 min. The absorption solution consists of 300 mg/L H_2_O_2_ (30% solution, Sigma-Aldrich, Schnelldorf, Germany) in 10 mL of MilliQ water. As an internal standard, 5 mg/L phosphate (1000 mg/L phosphate solution, Merck) was added.

### 2.4 Validation of the Analytical Method

To validate the accurateness and reliability of the developed method, PVC contents were determined by seven hard and soft PVC products from local hardware stores: sheet piling (dark and light), panel (gray and transparent), flexible tubing, corrugated roof panel, and pond liner. The PVC content of all analyzed products was determined gravimetrically as well as by ^1^H NMR.

For the gravimetric analysis, 0.5 g of each product was weighed and dissolved in THF. Each solution was then filtered to remove possible insoluble inorganic components. An excess of methanol was added to the filtrate to precipitate PVC, while organic additives such as phthalates were still dissolved. The solutions were then centrifuged, and the supernatant was decanted. This complete cleaning process was repeated 3 times for each product. After the solvent has been evaporated, the cleaned products were weighed and the PVC content was determined.

^1^H NMR spectra were recorded on a Magritek 80 MHz Carbon Ultra spectrometer (Magritek, Aachen, Germany) at ambient temperature. The chemical shifts were given in ppm (parts per million) and were referenced to the residual ^1^H-signal of the solvent (δ_H_ 3.58 regarding −CH_2_–(2,5) of THF-d8). 0.5 g of each PVC product was weighed in a glass vial and dissolved in 0.5 mL of THF-d8. Thereafter, samples were filtered using a glass fiber filter (FT-3-1102-050; Grade: MGB; Sartorius Lab Instruments GmbH & Co. KG, Göttingen, Germany), and the filtrate was transferred to an NMR tube. For quantification, a sample of dimethyl terephthalate (*c* = 0.051 mol/L, THF-d8) was used as an external standard. The methyl group singlet (6H, 4.05–3.75 ppm) was used as reference resonance for quantification. Regarding the PVC quantification, the resonance of the −CHCl– group between 4.90 and 4.10 ppm was integrated and calculated in comparison to the external standard. Each resonance was divided in comparison to the integral of the residual proton signal of THF-d8 (−CH_2_–(2,5), 3.62–3.54 ppm) as an internal standard. In the cases of the flexible tubing and the pond liner, the PVC signal was corrected of superimposing impurities using the fitting tool of MestReNova version 14.1.0-24037.

To elucidate possible interferences, the influence of chlorine-containing substances and organic as well as inorganic matrix components was tested. For this purpose, a synthetic sample containing inorganic and organic chlorine was prepared. As an inorganic chlorine source, 25 mg of sodium chloride (Merck) was added. As chlorine-containing organic substances, sucralose (Toronto Research Chemicals, Toronto, Canada), hydroxychloroquine (Sigma-Aldrich), diclofenac (Sigma-Aldrich), chloramphenicol (Sigma-Aldrich), triclosan (Sigma-Aldrich), and a chlorobiphenyl mixture (PCB 28, PCB 31, PCB 52, PCB 77, PCB 101, PCB 105, PCB 118, PCB 126, PCB 128, PCB 138, PCB 153, PCB 156, PCB 169, PCB 170, PCB 180; Sigma-Aldrich) were added. Concentrations equivalent to a chlorine content of 0.1 mg/g PVC were chosen for each chlorine-containing substance. Additionally, 1.0 mg of chloroprene rubber from a water sports sock (kitefly.de, Leipzig, Germany) was used as a possible interfering polymer. To simulate the effects of organic matrices, various plant components were investigated, such as leaves and acorns from European oak (*Quercus robur*), leaves from lily of the valley (*Convallaria majalis*), Japanese quince (*Chaenomeles japonica*), and European yew (*Taxus baccata*). All plant components were separately freeze-dried and milled using a planet mill (Fritsch, Idar-Oberstein, Germany). In total, 5 mg of each plant component was used for each batch. The synthetic samples and natural materials were extracted and measured using the methodology described above.

### 2.5 Environmental Samples

SPM samples were taken from the Rhine river (kilometer 590.4; Koblenz, Germany) using a filter net (300 μm mesh size, HydroBios, Altenholz, Germany) at 70 cm depth for 1 h on three different days (filtered volume ranging between 196–228 m^3^, Table S7) and a flow centrifuge (Carl Padberg/CEP, Lahr, Germany) for 1 to 5 h on eight different days with a flow rate of 17.8 L/min (sampled volume ranging between 110–558 m^3^). After sampling, all samples were separated into three fractions (5–1 mm, 1 mm to 500 μm, 500–300 μm (filter net) and 500–100, 100–50, 50–10 μm (flow centrifuge)), freeze-dried, and milled.

Sediment samples were taken from a harbor basin of the river Main (kilometer 280.5; Germany). All samples were separated into three size fractions (500–200, 200–50, 50–10 μm) and dried. For analyzing environmental samples, 1.0 g of sediment or SPM was weighed into the extraction cells, extracted, and analyzed as described above.

### 2.6 Quality Assurance and Quality Control

Only PVC-free sampling devices and PVC-free laboratory equipment were used. To prevent contaminations, it was further essential to wear only cotton clothes and safety clothing, which are free of PVC. All glassware and laboratory equipment used were cleaned prior to each step with ethanol (HPLC grade, Merck). All extraction cells were precleaned with bidest water and acetone (HPLC grade, Merck) and heated for 1 h at 100 °C. Collection vials were cleaned using acetone as well. All glass fiber filters and silica gel were heated in a muffle furnace at 450 °C for 2 h. Also, sea sand (600 °C for 2 h) and C-IC ceramic boats (1100 °C for 1 h) were heated. Furthermore, sea sand blanks (*n* = 8) were extracted and analyzed to determine the background PVC contamination. In addition, full procedural blanks were performed in parallel to each sample series to identify and quantify secondary contaminations and to confirm the cleanliness of the extraction system (extraction cells filled with sea sand) and the C-IC device (empty ceramic boats).

## 3 Results and Discussion

### 3.1 Calibration and Recovery

**Figure 1:**
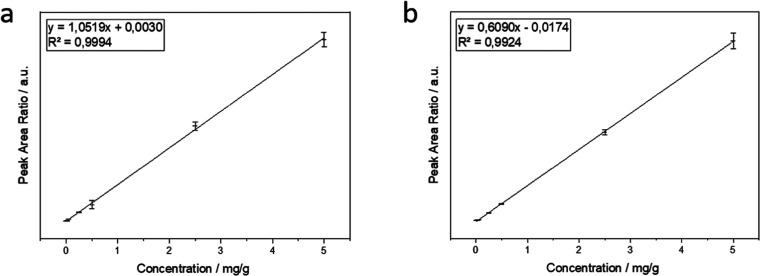
(a) Calibration curve for direct PVC combustion. (b) Calibration curve for PVC combustion after extraction. Calibration range was 0.005–5.0 mg/g.

The calibration *via* direct combustion of PVC as well as the calibration with PLE using a methanol cleanup and THF extraction showed a very good linearity over the whole concentration range, indicated by correlation coefficients of *R*^2^ = 0.9994 and *R*^2^ = 0.9924, respectively (Figure 1). Recoveries of 91.9 ± 5.5% (*n* = 8) in heated sea sand in comparison to calibration with direct PVC analysis and 85.5 ± 11.5% (*n* = 8) in EBS sediment in comparison to calibration with PVC combustion after extraction indicated the excellent efficiency of the procedure (Tables S2–S4).

### 3.2 Limit of Quantification

Using calibration over direct PVC combustion, the instrumental detection limit of the C-IC system was determined as 5.4 μg/g. However, due to the omnipresence of PVC in laboratories and rooms, the quantification is limited by the background concentration of PVC and not by the sensitivity of the C-IC system. Therefore, eight blank samples consisting exclusively of calcined sea sand were processed as the other samples. Based on these data, the mean blank PVC concentration with the two-sided confidence interval (CI) 95% (*t*_(*p*=0.95;*n*–1=7)_ = 2.365) was calculated. The limit of quantification (LOQ) was defined as the upper CI limit plus 2·σ. Or in other words, the LOQ was defined as the PVC concentration at which 97.5% of blank samples show a lower signal. This resulted in a LOQ of 8.3 μg/g for the PVC method *via* PLE combined with C-IC. A concentration of 7.6 ± 0.2 μg/g PVC was determined as background contamination (*n* = 8, Table S1).

### 3.3 Recoveries for Various PVC Products

Since there are plenty of products containing PVC, a large variety of PVC formulations are used. To test the influence of the formulation on the extraction efficiency, recoveries for different soft and hard PVC products were determined. For comparison, the PVC contents of products were determined gravimetrically and *via* NMR. Since the results of both methods were in good accordance (Table S5), the mean of both methods was used to determine the recoveries. Recoveries were calculated using the following formula
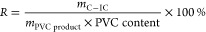
with *m*_C-IC_ is the PVC mass determined *via* C-IC after PLE, *m*_PVC product_ is the mass of PVC product, and PVC content is the PVC content of the particular PVC product.

Table 2 lists the recoveries for the different PVC products using C-IC after PLE. In the PVC products, the recoveries ranged from 97.8 to 109% with a mean of 103 ± 4%. Obviously, the extraction efficiencies were quantitative and independent of PVC formulations. Therefore, the used PVC calibration standard was appropriate for the selected PVC products.

**Table 2:** List of All PVC Products Examined, the Average PVC Content Determined *via* Gravimetrically, NMR, and the Determined Recoveries

PVC product	PVC content (%)	recovery (%)
sheet piling (dark)	85.4	97.8
sheet piling (light)	82.1	99.2
panel (gray)	89.0	101
panel (transparent)	98.7	101
corrugated roof panel	103	104
flexible tubing	66.6	107
pond liner	54.6	109

### 3.4 Analysis of Synthetic Samples

To evaluate the selectivity of the method, a synthetic sample containing inorganic and organic chlorinated compounds was analyzed. No chloride signal above the LOQ was detected *via* C-IC. Thus, chlorinated organic compounds such as PCBs, diclofenac, and triclosan were quantitatively removed in the methanol extraction step. Inorganic compounds such as sodium chloride are insoluble in organic solvents and are therefore not extracted with THF. The same is true for chloroprene rubber, which could be observed due to the fact that the polymer pieces were present in the extraction cell in an optically unchanged form even after the complete extraction process. To test the influence of matrix ingredients, plant material (free of PVC) was extracted and analyzed. With all of these matrices, chloride was not detected *via* C-IC, and therefore, we received no PVC blank values.

We observed that none of the chlorinated substances and plant components had an interfering effect on the C-IC measurement, and therefore, no influence on the analysis of PVC was observed. Thus, this method is selective for chlorine-containing polymers and is not limited by matrix effects known for other thermoanalytical methods.^6,19,24,25,27,28^

### 3.5 Environmental Samples

**Figure 2:**
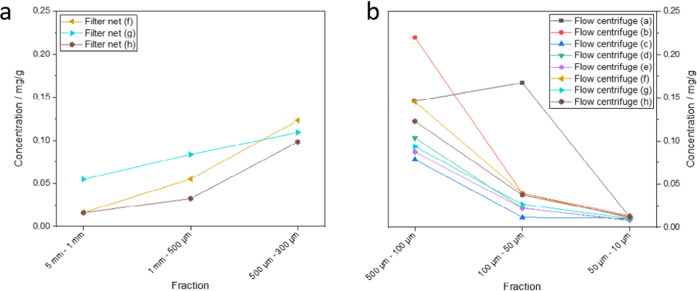
PVC concentrations in suspended matter of the river Rhine obtained with filter nets (a) and a flow centrifuge (b).

Sediments and SPM of the river Rhine were analyzed with the optimized analytical method with regard to their PVC contamination. In all SPM samples collected from the Rhine at Koblenz (590.4 km), PVC was detected above LOQ of 8.3 μg/g. The observed concentrations ranged from 8.3–220 μg/g. The highest concentration levels were found in medium-sized fractions (100–500 μm). This was observed with filter nets (300–500 μm) and a flow centrifuge (100–500 μm) (Figure 2). In comparison to the medium-sized fractions, the PVC concentrations decreased continuously down to a particle size of 10 μm and up to a particle size of 5 mm (Table S6). Thus, primary or secondary PVC microplastics are predominantly present in Rhine in SPM at a particle size of 100–500 μm. However, further detailed studies are needed to underline those observations.

**Figure 3:**
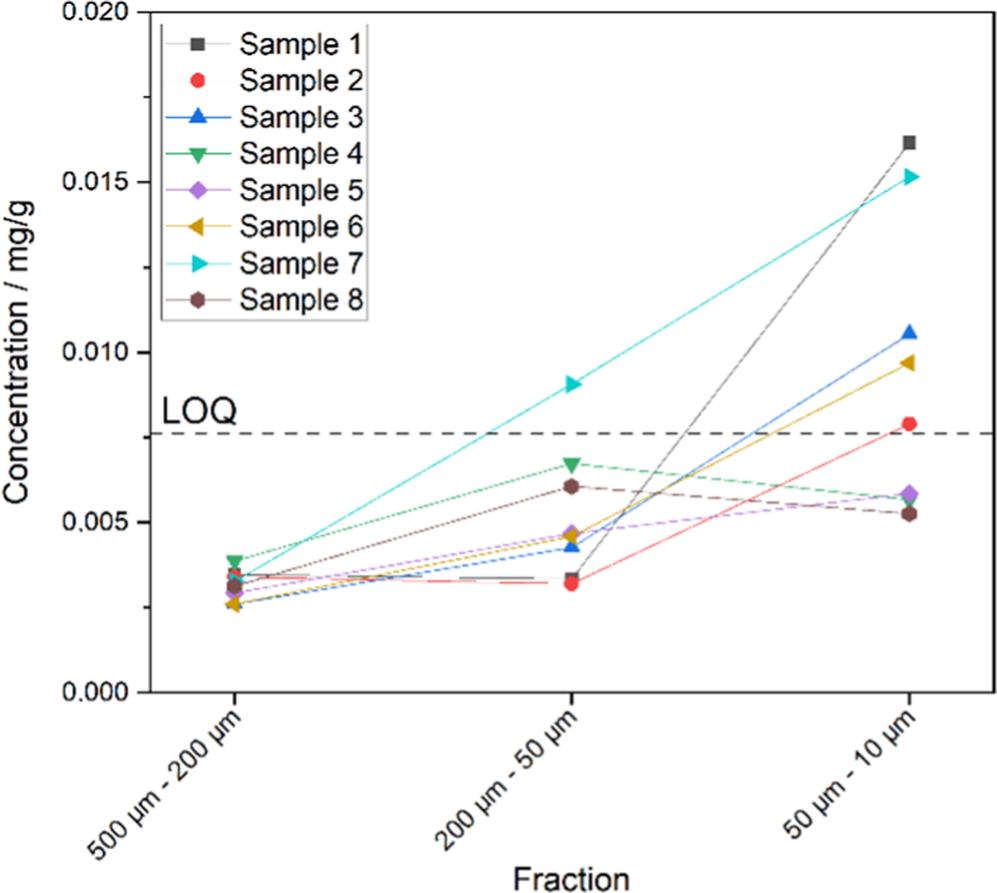
Results of sediment samples from a habor basin of the river Main (concentration *vs* fraction size). It can be seen that the PVC concentration is mostly highest in the smallest fraction (10–50 μm).

In contrast to the results of SPM from the river Rhine, the maximum concentrations in the sediments from the harbor basin of the river Main (280.5 km) were found in the fraction with the smallest particle sizes ranging from 10–50 μm (Figure 3 and Table S8). With increasing particle sizes, the PVC concentration levels were declining. PVC was not detected above LOQ in all samples in the fraction with the particle size ranging from 200–500 μm. Since in the harbor basin, there are calmer waters compared to the river Main coupled with the high density of PVC, a high sedimentation rate can be expected. So, the small PVC concentrations measured assume an overall small PVC contamination in the sampled area. The developed method can be used in further studies regarding the occurrence and distribution of PVC in surface waters and soil.

However, it has to be noted that this analytical method can only be used to determine the mass of the polymer itself and not of the PVC particles consisting of additives and fillers. Since soft PVC products contain plasticizers up to 50% of the product mass, the gap between polymer masses and product masses could be significant in comparison to other polymers, such as PP.

A possible limitation of the developed method might be a negative influence of the matrix caused by premature HCl loss during the MeOH extraction. However, this is rather unlikely since our recoveries of PVC were quantitative and we found no indications underlining this assumption. Furthermore, the results of the described method should be assessed as a sum of all chlorine-containing polymers, which are extractable under the chosen conditions. In addition to PVC (chlorine content 56%), chlorinated polyethylene (chlorine content 34–44%) or chlorinated PVC (chlorine content 62–69%) might be present. However, the overdetermination of “normal” PVC masses should be rather small due to the marginal differences in the chlorine contents and a much higher production volume of PVC compared to chlorinated polyethylene and chlorinated PVC. Thus, the developed method provides reliable concentrations for the sum of chlorine-containing polymers, expressed as PVC concentrations. Finally, silica gel extracts can be also used for parallel quantification of PE, PS, and PP using the pyrolysis-GC-MS method as described by Dierkes et al.^13^

## Supporting Information Available

Details about the experimental setup and PVC concentrations for all measured samples and sampling parameters (PDF)
