# Supplementary material for: Quantification of Poly(vinyl chloride) Microplastics via Pressurized Liquid Extraction and Combustion Ion Chromatography
Source: Environ Sci Technol. 2023 Mar 14;57(12):4806–12. doi: 10.1021/acs.est.2c06555 (PMC10061920; doi:10.1021/acs.est.2c06555)
Supplement: Supplementary file 1 — es2c06555_si_001.pdf [file es2c06555_si_001.pdf]

Supporting Information

Quantification of polyvinyl chloride microplastics *via* pressurized liquid extraction and combustion ion chromatography

Jan Kamp[1,2], Georg Dierkes[1], Peter Nikolaus Schweyen[1], Arne Wick[1], Thomas A. Ternes[1,2]*

[1]Federal Institute of Hydrology, Department G2 – Water Chemistry, Am Mainzer Tor 1, 56068, Koblenz, Germany

[2]University of Koblenz · Landau, Universitätsstraße 1, 56070, Koblenz, Germany

*To whom correspondence should be addressed: ternes@bafg.de

Contents

Figure S. 1: Experimental set-up and process from the sample extraction to the combustion ion chromatography measurement....S2

Table S. 1: Determined PVC background concentrations of sea sand blank samples. S2

Table S. 2: Determined PVC concentrations and recoveries of the spiked sea sand samples. S3

Table S. 3: Determined PVC concentrations of the blank EBS sediments....S3

Table S. 4: Determined PVC concentrations and recoveries of the spiked (1.0 mg/g PVC) EBS sediment. S4

Table S. 5: List of all PVC products examined and the PVC content determined via C-IC, gravimetrically and NMR. S4

Table S. 6: List of all suspended matter samplings with fractions, sampling days and determined PVC concentrations....S5

Table S. 7: List of all sampling parameters of the suspended matter samplings. S6

Table S. 8: List of all sediment samples with fractions and determined PVC concentrations. S7

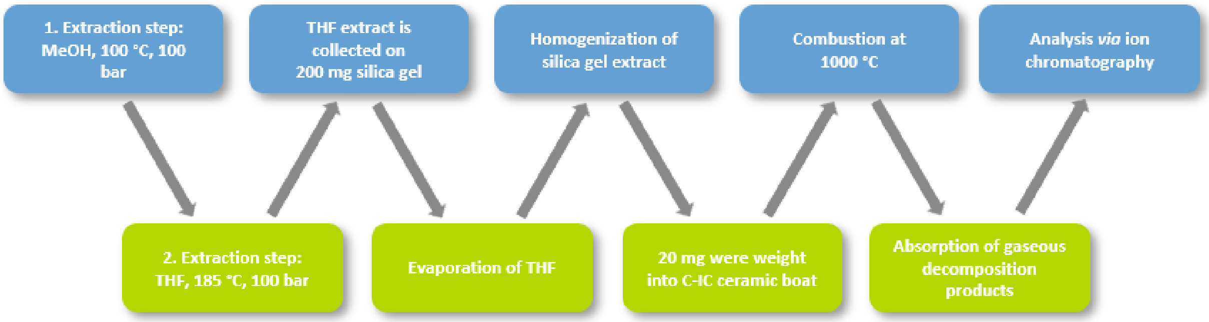

Figure S. 1: Experimental set-up and process from the sample extraction to the combustion ion chromatography measurement.

Table S. 1: Determined PVC background concentrations of sea sand blank samples.

Sample	PVC conc. (C-IC) / µg/g	Average PVC concentration / µg/g
Sea sand blank 1	7,9	
Sea sand blank 2	7,8	
Sea sand blank 3	7,9	
Sea sand blank 4	7,6	
Sea sand blank 5	7,6	7,6 ± 0,3
Sea sand blank 6	7,3	
Sea sand blank 7	7,3	
Sea sand blank 8	7,3	

Table S. 2: Determined PVC concentrations and recoveries of the spiked sea sand samples.

Sample	PVC conc. (C-IC) / mg/g	PVC recovery / %	Average PVC recovery / %
Sea sand 1	2.078	83.12	
Sea sand 2	2.170	86.80	
Sea sand 3	2.209	88.36	
Sea sand 4	2.378	95.12	91.92 ± 5.50
Sea sand 5	2.323	92.92	
Sea sand 6	2.343	93.72	
Sea sand 7	2.511	100.4	
Sea sand 8	2.371	94.84	

Table S. 3: Determined PVC concentrations of the blank EBS sediments.

Sample	PVC conc. (C-IC) / mg/g	Average PVC conc. / mg/g
EBS sediment blank 1	0.034	
EBS sediment blank 2	0.037	
EBS sediment blank 3	0.034	
EBS sediment blank 4	0.044	0.020 ± 0.019
EBS sediment blank 5	0.003	
EBS sediment blank 6	0.003	
EBS sediment blank 7	0.003	
EBS sediment blank 8	0.003	

Table S. 4: Determined PVC concentrations and recoveries of the spiked (1.0 mg/g PVC) EBS sediment.

Sample	PVC conc. (C-IC) / mg/g	PVC recovery / %	PVC recovery (blank correction) / %
EBS sediment 1	0.972	97.20	95.19
EBS sediment 2	0.953	95.30	93.29
EBS sediment 3	0.887	88.70	86.69
EBS sediment 4	1.009	100.9	98.89
EBS sediment 5	0.647	64.70	62.69
EBS sediment 6	0.794	79.40	77.39
EBS sediment 7	0.875	87.50	85.49
EBS sediment 8	0.864	86.40	84.39

Table S. 5: List of all PVC products examined and the PVC content determined via C-IC, gravimetrically and NMR.

PVC product	PVC content (C-IC) / %	PVC content (gravimetric) / %	PVC content (NMR) / %
Sheet piling (dark)	85,4	89,4	85,3
Sheet piling (light)	82,1	80,9	84,6
Panel (grey)	89,0	87,6	87,9
Panel (transparent)	98,7	97,4	97,3
Corrugated roof panel	103	100	98,2
Flexible tubing	66,6	57,5	66,5
Pond liner	54,6	51,0	49,6

Table S. 6: List of all suspended matter samplings with fractions, sampling days and determined PVC concentrations.

Sampling technique	Fraction	Sampling day	PVC concentration / mg/g
Flow centrifuge	500 – 100 µm	a	0.146
	100 – 50 µm		0.167
	50 – 10 µm		0.011
Flow centrifuge	500 – 100 µm	b	0.220
	100 – 50 µm		0.039
	50 – 10 µm		0.013
Flow centrifuge	500 – 100 µm	c	0.079
	100 – 50 µm		0.012
	50 – 10 µm		0.011
Flow centrifuge	500 – 100 µm	d	0.103
	100 – 50 µm		0.022
	50 – 10 µm		0.008
Flow centrifuge	500 – 100 µm	e	0.087
	100 – 50 µm		0.022
	50 – 10 µm		0.009
Flow centrifuge	500 – 100 µm	f	0.145
	100 – 50 µm		0.039
	50 – 10 µm		0.011
Flow centrifuge	500 – 100 µm	g	0.093
	100 – 50 µm		0.027
	50 – 10 µm		0.010
Flow centrifuge	500 – 100 µm	h	0.123
	100 – 50 µm		0.037
	50 – 10 µm		0.012
Filter net	1 – 5 mm	f	0.016
	1 mm – 500 µm		0.055
	500 – 300 µm		0.123
Filter net	1 – 5 mm	g	0.055
	1 mm – 500 µm		0.084
	500 – 300 µm		0.109
Filter net	1 – 5 mm	h	0.016
	1 mm – 500 µm		0.032
	500 – 300 µm		0.098

Table S. 7: List of all sampling parameters of the suspended matter samplings.

Sampling technique	Sampling day	Sampling date	Turbidity / TE/F	Sampled volume / m³
Flow centrifuge	a	29.07.2020	4.37	5.54
	b	28.01.2021	15.5	4.89
	c	01.02.2021	140	1.10
	d	05.02.2021	56.0	3.31
	e	11.02.2021	33.0	3.01
	f	02.03.2021	6.30	5.58
	g	04.03.2021	5.60	5.23
	h	18.03.2021	7.90	5.19
Filter net	f	02.03.2021	6.30	212
	g	04.03.2021	5.60	196
	h	18.03.2021	7.90	229

Table S. 8: List of all sediment samples with fractions and determined PVC concentrations.

Sediment sample	Fraction	PVC concentration / mg/g
1	500 – 200 µm	0.003 (< LOQ)
	200 – 50 µm	0.003 (< LOQ)
	50 – 10 µm	0.016
2	500 – 200 µm	0.003 (< LOQ)
	200 – 50 µm	0.003 (< LOQ)
	50 – 10 µm	0.008
3	500 – 200 µm	0.003 (< LOQ)
	200 – 50 µm	0.004 (< LOQ)
	50 – 10 µm	0.011
4	500 – 200 µm	0.004 (< LOQ)
	200 – 50 µm	0.007 (< LOQ)
	50 – 10 µm	0.006 (< LOQ)
5	500 – 200 µm	0.003 (< LOQ)
	200 – 50 µm	0.005 (< LOQ)
	50 – 10 µm	0.006 (< LOQ)
6	500 – 200 µm	0.003 (< LOQ)
	200 – 50 µm	0.005 (< LOQ)
	50 – 10 µm	0.010
7	500 – 200 µm	0.003 (< LOQ)
	200 – 50 µm	0.009
	50 – 10 µm	0.015
8	500 – 200 µm	0.003 (< LOQ)
	200 – 50 µm	0.006 (< LOQ)
	50 – 10 µm	0.005 (< LOQ)
